# Molecular characteristics of the first case of haloxyfop-resistant *Poa annua*

**DOI:** 10.1038/s41598-020-61104-0

**Published:** 2020-03-06

**Authors:** H. Ghanizadeh, C. H. Mesarich, K. C. Harrington

**Affiliations:** School of Agriculture and Environment, Massey University, PB 11-222, Palmerston North, 4442 New Zealand

**Keywords:** Plant sciences, Plant ecology, Plant evolution, Plant stress responses

## Abstract

Haloxyfop is one of two acetyl-coenzyme A carboxylase (ACCase) inhibitors that is recommended for controlling *Poa annua*. We have characterised a population of *P. annua* that had developed resistance to haloxyfop. This resistant population was found to be almost 20 times less sensitive to haloxyfop than a susceptible population based on percentage survival of individuals in two dose-response experiments. However, the haloxyfop-resistant population was still susceptible to clethodim. Pre-treatment of resistant individuals with a cytochrome P450 inhibitor, malathion, did not change the sensitivity level of the resistant plants to haloxyfop, suggesting that a non-target site mechanism of resistance involving enhanced metabolism, was not responsible for this resistance in *P. annua*. Gene sequencing showed that a target site mutation at position 2041, which replaced isoleucine with threonine in the carboxyltransferase (CT) domain of the ACCase enzyme, was associated with resistance to haloxyfop in the resistant population. An evaluation of the 3-D structure of the CT domain suggested that, unlike Asn-2041, which is the most common mutation at this position reported to date, Thr-2041 does not change the conformational structure of the CT domain. This is the first study investigating the molecular mechanism involved with haloxyfop resistance in *P. annua*.

## Introduction

*Poa annua*, an annual C_3_ weedy grass in both agricultural and urban systems^1,2^, is one of the world’s most widely distributed plant species^2^. It is particularly problematic in turf grass, where it is highly tolerant of disturbance and has high fecundity^3^. The occurrence of *P. annua* is often associated with human activities, and is one of the weedy grass species that is often found in seedbanks^4^. *P. annua* is a prolific annual weed with a very short life^2^. Notably, it can produce seed throughout most of the year^1^. Collectively, these features predispose *P. annua* to herbicide resistance, since, at any one time, a large number of individuals will experience selection pressure under intense herbicide use^5^. *P. annua* is an allotetraploid (2n = 4 ×  = 28) species, originating from the hybridization of *P. supina* as the male parent and *P. infirma* as the female parent^6^.

Cultural practices that can help desirable species compete well with *P. annua* can be used for management of this weedy grass species in turf grasses^7^; i.e. in an effort to stop it re-establishing from seed after it completes its life cycle. Techniques such as soil aerification, fertilization management and modifying irrigation regimens are useful cultural practices for *P. annua* management in turf^8^. However, cultural techniques should be part of a management package^9^, as these techniques do not result in complete control of *P. annua* in turf. Indeed, application of herbicides is desirable for complete management of this weed species in turf^10^. There are, however, only a limited number of herbicides that can selectively remove *P. annua* from turf grasses^11^. Hence, the best strategy to manage *P. annua* in turf involves a combination of both cultural and chemical options^12^.

ACCase (acetyl-coA carboxylase) inhibitors are post-emergence herbicides that selectively control weedy grass species within broadleaf crops^13^. The ACCase reaction occurs in two enzymatic steps^14^, with the first step involving biotin carboxylase catalysing the carboxylation of biotin. In the second step, the carboxyl group from biotin is transferred to acetyl-CoA by carboxyltransferase (CT) to generate malonyl-CoA. ACCase inhibitors bind to the CT domain of the ACCase enzyme, and disrupt fatty acid synthesis in weedy grass species^15^. The selectivity of ACCase-inhibitors between weedy grass and broadleaf species stems from the differential forms of ACCase existing in the plastids of broadleaf plant species compared with weedy grass species. Grass species have a eukaryotic (rather than prokaryotic) form of ACCase that is sensitive to ACCase inhibitors^16^. ACCase-inhibitors can be divided into three classes, namely the aryloxyphenoxypropionates (APP), the phenylpyrazolines (PPZ) and the cyclohexanediones (CHD). So far, 12 APP, one PPZ and eight CHD herbicides are commercially available for controlling weedy grass species^17^.

Continuous applications of herbicides with the same mode of action can result in herbicide-resistant populations of weedy plant species^18,19^. Both target site^20^ and non-target site^21^ mechanisms can cause resistance to herbicides in weed species. In the target site mechanism of resistance, amino acid substitutions at positions Trp-1999, Trp-2027, Ile-2041, Gly-2096, Ile-1781, Asp-2078 and Cys-2088 have been identified from ACCase-inhibitor-resistant weedy grass species^13^. According to Beckie and Tardif^22^, the pattern of resistance to the three classes of ACCase inhibitors is varied among weedy plant species, and is dependent on the position of mutations and amino acid substitutions. The non-target site mechanism of resistance to ACCase-inhibitors in weedy grass species involves enhanced herbicide metabolism, mainly through the enzyme cytochrome P450^21^. *P. annua* has an inherent tolerance to most ACCase-inhibitors, due to a fixed Leu residue at position 1781, which results in an insensitive form of ACCase^23^. It has been hypothesised that *P. annua* inherited this inherent tolerance from *P. supina*^23^. However, clethodim (CHD) and haloxyfop (APP) are two ACCase-inhibitors that do provide effective control of *P. annua*^24^.

Recently, it was reported that haloxyfop failed to control *P. annua* in a New Zealand golf course^25,26^. The golf course had a *Festuca rubra* turf, and this species has an inherent tolerance of all ACCase-inhibitors, including haloxyfop^23^. Hence, haloxyfop had been used to remove sensitive weedy grass species such as *P. annua* safely from the *F. rubra* turf. However, a population of *P. annua* was reported to have built up resistance to haloxyfop after frequent applications of haloxyfop^25^. The recommended rate of haloxyfop was no longer able to effectively control this weedy grass species. Preliminary experiments confirmed that the suspected resistant plants could survive 130 g ae ha^−1^ of haloxyfop compared with putative susceptible plants that were completely controlled by this rate of haloxyfop^25^. To date, resistance to EPSP synthase inhibitors, ALS inhibitors, photosystem II inhibitors, microtubule inhibitors, carotenoid biosynthesis, PSI electron diverters and lipid inhibitors in *P. annua* has been reported worldwide^17^. However, to our knowledge, this is the only case of evolved haloxyfop resistance in *P. annua* reported to date^17^. Here, we have assessed the level of resistance to haloxyfop and the pattern of cross-resistance to the other recommended ACCase-inhibitor, clethodim. We have also evaluated the molecular basis of resistance to haloxyfop in this haloxyfop-resistant *P. annua*.

## Results

### The response of populations to haloxyfop

**Figure 1:**
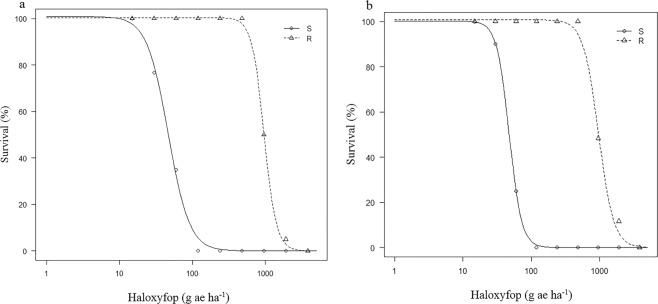
Fitted haloxyfop dose-response curves (on a logarithmic dose scale) for two *P. annua* populations, the resistant population R and the susceptible population S in (**a**) Experiment 1 and (**b**) Experiment 2. The fitted curves were produced using the survival of treated plants as a percentage of untreated control.

The results for the first and second runs of haloxyfop dose-response experiments are illustrated in Fig. 1a,b. In the first run, 100% survival was recorded for the haloxyfop-resistant population (R) when treated with haloxyfop application rates up to 480 g ae ha^−1^, while 100% mortality was observed for the individuals of the haloxyfop-susceptible population (S) when treated with 120 g ae ha^−1^ of haloxyfop. Similar results were obtained for the second run of this dose-response experiment. The values for 50% reduction in survival of individuals (LD_50_) were estimated to be 962.2 and 46.4 g ae h^−1^ for the populations R and S, respectively, in the first run of the haloxyfop dose-response experiment (Table 1). The values for 50% reduction in dry weight of individuals (GR_50_) for the populations R and S were estimated to be 366.8 and 19.3 g ae ha^−1^. Based on LD_50_ R/S (resistant/susceptible) and GR_50_ R/S ratios, it was estimated that population R was 20.7- and 19.0-times less sensitive to haloxyfop, respectively, than population S. Similar results were obtained in the second run of this dose-response experiment (Table 1).

**Table 1:** Parameters (see footnote) estimated from the nonlinear regression analysis of haloxyfop dose–response experiments for haloxyfop-resistant (R) and susceptible (S) populations at 28 days after treatment.

First Experiment				
**Survival**				
Population	b(SE)	d(SE)	LD_50_(SE)	LD_50_RF
S	2.9 (0.2)	100.9 (1.8)	46.4 (1.5)	20.7
R	5.2 (0.9)	100.4 (0.9)	962.2 (16.4)	
**Dry weight**				
Population	b(SE)	d(SE)	GR_50_(SE)	GR_50_RF
S	1.9 (0.3)	100.0 (4.5)	19.3 (1.8)	19.0
R	1.1 (0.1)	101.1 (3.6)	366.8 (47.4)	
**Second Experiment**				
**Survival**				
Population	b(SE)	d(SE)	LD_50_(SE)	LD_50_RF
S	4.8 (0.4)	100.1 (1.3)	47.6 (1.1)	20.3
R	3.9 (0.3)	100.8 (0.7)	964.3 (17.9)	
**Dry weight**				
Population	b(SE)	d(SE)	GR_50_(SE)	GR_50_RF
S	1.9 (0.2)	99.9 (3.6)	24.0 (1.7)	15.3
R	1.3 (0.1)	103.2 (2.3)	366.2 (29.7)	

d = the upper limit, b = the slope around the LD50 or GR50, SE = standard error, LD50 = the rate of herbicide (g ae ha-1) required to 50% mortality, GR50 = the rate of herbicide (g ae ha-1) required to reduce shoot dry weight by 50%, LD50 RF = resistant/susceptible factor based on LD50 ratios, GR50 RF = resistant/susceptible factor based on GR50 ratios.

### The response of populations to haloxyfop plus malathion

The responses of the individuals of both populations when treated with malathion prior to haloxyfop applications was similar to those that were only treated with haloxyfop (Tables 1 and 2). The LD_50_ values in the first haloxyfop plus malathion dose-response experiment were 919.6 and 49.4 g ae ha^−1^ for populations R and S, respectively, resulting in population R being 18.6 times more resistant to haloxyfop than population S. The second run showed an 18.5-fold difference for LD_50_. Likewise, application of malathion prior to haloxyfop also resulted in GR_50_ values similar to when no malathion was used. Population R was 16.7-fold more resistant to haloxyfop plus malathion than population S, based on the GR_50_ R/S ratio in the first run, and a 21.8-fold difference was recorded in the second run (Table 2).

**Table 2:** Parameters (see footnote) estimated from the nonlinear regression analysis of haloxyfop plus malathion dose–response experiments for haloxyfop-resistant (R) and susceptible (S) populations at 28 days after treatment.

First Experiment				
**Survival**				
Population	b(SE)	d(SE)	LD_50_(SE)	LD_50_RF
S	4.4 (0.4)	100.2 (1.6)	49.4 (1.7)	18.6
R	4.5 (0.6)	100.7 (0.9)	916.6 (17.9)	
**Dry weight**				
Population	b(SE)	d(SE)	GR_50_(SE)	GR_50_RF
S	1.9 (0.2)	99.6 (2.9)	20.1 (3.2)	16.7
R	1.3 (0.1)	102.9 (1.9)	336.0 (68.1)	
**Second Experiment**				
**Survival**				
Population	b(SE)	d(SE)	LD_50_(SE)	LD_50_RF
S	3.3 (0.3)	100.6 (1.9)	49.9 (1.7)	18.5
R	4.7 (0.7)	100.6 (0.9)	927.8 (19.1)	
**Dry weight**				
Population	b(SE)	d(SE)	GR_50_(SE)	GR_50_RF
S	3.1 (0.4)	100.7 (3.2)	21.1 (2.4)	21.8
R	1.5 (0.1)	99.9 (1.9)	459.7 (30.1)	

d = the upper limit, b = the slope around the LD50 or GR50, SE = standard error, LD50 = the rate of herbicide (g ae ha-1) required to 50% mortality, GR50 = the rate of herbicide (g ae ha-1) required to reduce shoot dry weight by 50%, LD50 RF = resistant/susceptible factor based on LD50 ratios, GR50 RF = resistant/susceptible factor based on GR50 ratios.

### The response of populations to clethodim

**Figure 2:**
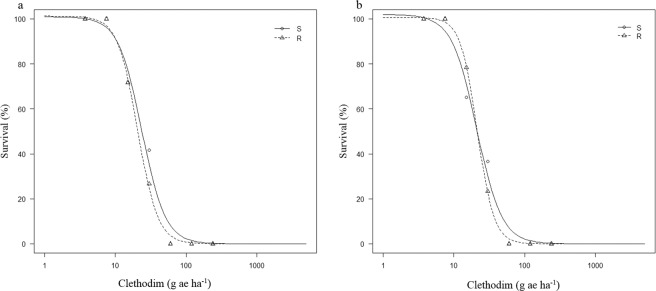
Fitted clethodim dose-response curves (on a logarithmic dose scale) for two *P. annua* populations, the resistant population R and the susceptible population S in (**a**) Experiment 1 and (**b**) Experiment 2. The fitted curves were produced using the survival of treated plants as a percentage of untreated control.

Both of the clethodim dose-response experiments showed that the haloxyfop-resistant population was still susceptible to clethodim (Fig. 2a,b). At 120 g ae ha^−1^ (recommended label rate) of clethodim, 100% mortality was recorded for individuals of both populations R and S. The LD_50_ values for populations R and S were similar and ranged from 20 to 23 g ae ha^−1^ in the first and second clethodim dose-response experiments (Table 3). Likewise, the GR_50_ values of both R and S populations were similar and ranged from 13 to 16 g ae ha^−1^ in the first and second clethodim dose-response experiments (Table 3). Thus, these results indicated that there was no cross-resistance to clethodim in the haloxyfop-resistant population (R) of *P. annua* studied in this research.

**Table 3:** Parameters (see footnote) estimated from the nonlinear regression analysis of clethodim dose–response experiments for haloxyfop-resistant (R) and susceptible (S) populations at 28 days after treatment.

First Experiment				
**Survival**				
Population	b(SE)	d(SE)	GR_50_(SE)	LD_50_RF
S	2.6(0.3)	100.9(3.0)	23.6(1.6)	0.9
R	3.1(0.4)	101.3(2.9)	20.7(1.2)	
**Dry weight**				
Population	b	d	GR_50_	GR_50_RF
S	2.5(0.5)	99.7(4.6)	13.2(1.2)	1.1
R	2.6(0.5)	100.7(4.2)	14.7(1.2)	
**Second Experiment**				
**Survival**				
Population	b(SE)	d(SE)	LD_50_(SE)	LD_50_RF
S	2.5(0.3)	101.9(2.8)	21.0(1.3)	1.0
R	3.7(0.5)	100.6(2.5)	21.4(1.1)	
**Dry weight**				
Population	b(SE)	d(SE)	GR_50_(SE)	GR_50_RF
S	2.4(0.3)	101.6(3.7)	15.2(1.1)	1.1
R	2.6(0.4)	100.9(3.3)	16.5(1.1)	

d = the upper limit, b = the slope around the LD_50_ or GR_50_, SE = standard error, LD_50_ = the rate of herbicide (g ae ha^−1^) required to 50% mortality, GR_50_ = the rate of herbicide (g ae ha-1) required to reduce shoot dry weight by 50%, LD_50_ RF = resistant/susceptible factor based on LD_50_ ratios, GR_50_ RF = resistant/susceptible factor based on GR_50_ ratios.

### *ACCase* sequencing

The partial amplification of the *ACCase* gene using the first primer pair, accf1/accr1, resulted in a 567-bp amplicon of that covers the mutation site 1781. The partial amplification of the *ACCase* gene showed that both the R and S populations have an amino substitution at position 1781 that results from an A to T change at the first codon of isoleucine, and this changes Ile to Leu (S1a and b). Also, it was observed that the sequence obtained from both populations using the first primer pair was polymorphic at codon 1781, since both T and A nucleotides presented at the same time.

The second primer pair, accf2/accr2, amplified a fragment of 762 bp from the *ACCase* gene covering the mutation sites 1999, 2027, 2041, 2096, 2078 and 2088. A subsequent sequence alignment revealed an amino acid substitution of Ile-2041-Thr for population R (Fig. S2a). This amino acid substitution resulted from a T to C change at the second codon of isoleucine. However, nucleotide polymorphisms were noted at site 2041 for population R, as two peaks were recorded at this position in the chromatogram for population R compared with population S (Fig. S2a,b). In order to further investigate the observed nucleotide polymorphism, the amplified fragment from the second primer pair was cloned into a plasmid and sequenced. The results showed that there were two distinct sequences for population R at the mutation site 2041 (Fig. S3a,b). One of the sequences carries the mutation (ACT) that results in the Ile-2041-Thr substitution, while the wild type sequence (ATT) was recorded for the other sequence.

### ACCase 3-D structure analysis

**Figure 3:**
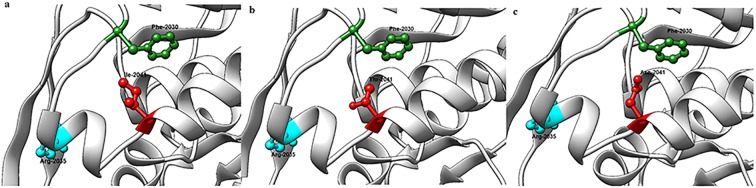
The 3-D view of the inside of the CT herbicide-binding site cavity for ACCase with (**a**) Ile-2041(wild-type), (**b**) Thr-2041 and (**c**) Asn-2041 amino acid residues.

**Figure 4:**
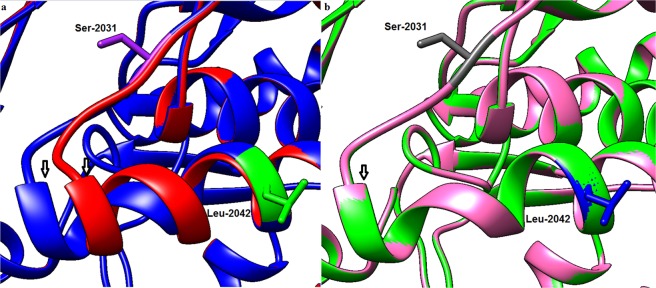
The effect of (**a**) Asn-2041 and (**b**) Thr-2041 mutations on the structure of herbicide-binding site of ACCase. The ribbon colours represent (**a**) red: the Asn-2041 (haloxyfop-resistant) and blue: Ile-2041(haloxyfop-susceptible); and in (**b**) pink: Thr-2041 (haloxyfop-resistant) and green: Ile-2041 (haloxyfop-susceptible). The arrows in Panel (a) show where structural torsion would occur for the Asn-2041 mutation. No structural torsion would occur for the Thr-2041(represented by merging of the pink and green colours and by a single arrow in Panel (b)).

The modelled 3-D structure of CT domains for different amino acid substitutions are illustrated in Fig. 3. The substitution of the amino acid isoleucine with either threonine or asparagine (the most common amino acid reported in other ACCase-inhibitor-resistant species to date) would result in a replacement of a hydrophobic amino acid that contains an aliphatic side chain with a polar amino acid. Although both asparagine and threonine are from the same class of amino acids, asparagine contains a carboxamide side chain, whereas threonine contains a side chain with a hydroxyl group. Asparagine has a gamma amino group but threonine has no amino moiety in its R group. According to the modelled 3-D structures for the CT domain, both Ile-2041 and Thr-2041 are oriented toward the opening of the CT domain active-site cavity (Fig. 3a,b). However, Asn-2041 is protruded towards the inside of the cavity of CT active site (Fig. 3c). The modelled 3-D structures in complex with haloxyfop showed that the Asn-2041 mutation would result in major conformational changes in the herbicide-binding site in the form of a severe torsion encompassing the amino acid Gly-2035 (Fig. 4a). However, no conformational changes in the herbicide-bonding site were noted for the Ile-2041-Thr substitution (Fig. 4b).

## Discussion

Resistance to commonly used herbicides such as EPSP synthase inhibitors, ALS inhibitors and photosystem II inhibitors has been reported in *P. annua* globally^17^. In this research, we confirmed and characterised the first case of resistance to an ACCase-inhibitor, haloxyfop, in *P. annua*. Currently, there are over 40 weed species that have evolved resistance to ACCase-inhibitors globally^17^. Resistance to ACCase-inhibitors appears to develop more frequently than to most other herbicides with different modes of action, and this is mainly because there are many potential amino acid substitutions that can confer resistance to at least one of the classes of ACCase-inhibitors^27^.

The dose-response experiments revealed that the haloxyfop-resistant population (R) was at least 20- and 15 times less sensitive to haloxyfop than the susceptible population (S), based on LD_50_ R/S and GR_50_ R/S, respectively. However, there was no cross-resistance to clethodim in population R. Both target site mutation and non-target site mechanisms can confer resistance to ACCase-inhibitors^21,27^. The enhanced herbicide metabolism of ACCase inhibitors has been recorded in several weedy grass species such as *Lolium rigidum*^28^, *Digitaria sanguinalis*^29^ and *Alopecurus myosuroides*^30^. An enzyme, cytochrome P450 monooxygenase (P450), is known to cause enhanced herbicide metabolism of ACCase-inhibitors in some resistant weedy grass species^21^. Malathion is an organophosphate insecticide that inhibits P450 activity^31^, and thus stops the enhanced metabolism of ACCase-inhibitors in these resistant weeds^32,33^. However, it appears that enhanced herbicide metabolism is not involved in resistance to haloxyfop in population R, as the individuals of this population that were treated with malathion prior to haloxyfop treatments showed no differences in response to haloxyfop compared to those that were only treated with haloxyfop.

In most cases of resistance to ACCase inhibitors, an insensitive ACCase, due to target site mutations, has been the mechanism of resistance^15,27^. So far, allelic variants at positions Ile-1781, Trp-1999, Trp-2027, Ile-2041, Asp-2078, Cys-2088 and Gly-2096 have been associated with resistance to ACCase-inhibitors^13^. In the research reported here, primer pairs accf1/accr1 and accf2/accr2 were used to amplify a region of 1255 bp in the *ACCase* gene (specifically, the region encoding the CT domain) containing all of the sites mentioned above. Sequencing and assembly of the amplified region using the first primer pair, accf1/acr1, in both populations R and S, identified a single amino acid substitution at position 1781. This nucleotide substitution at position 1781 results in a substitution of isoleucine to leucine (i.e. Ile-1781-Leu)^23^. However, it appears that this residue only exists in one of the homoeologous ACCase proteins. Similar results have been reported previously for *P. annua*^23,34^. It is known that *P. annua* is tolerant of some of the ACCase inhibitors, owing to the amino acid substitution at position Ile-1781 of the ACCase protein^13^. Haloxyfop and clethodim are the only ACCase-inhibitors registered in New Zealand for controlling *P. annua*. The amino acid Ile-1781 is located at the opening of the cavity of the CT domain^35^. Délye *et al*.^35^ suggested that some ACCase-inhibiting herbicides fitted better into the cavity of the chloroplastic ACCase CT domain active site, and this could account for the differences in ACCase sensitivity to some but not all APPs and CHDs. By using three-dimensional models, they also noted that the Ile-1781-Leu mutation did not significantly affect the sensitivity of ACCase to clethodim^35^. In *L. rigidum*, this Ile-1781-Leu mutation confers some levels of resistance to haloxyfop and clethodim, though only individuals that were homozygous for Ile-1781-Leu appeared to be resistant to clethodim^36^. In addition, Guo *et al*.^37^ noted that the Ile-1781-Leu mutation made *Alopecurus aequalis* 8- and 9-fold more resistant to haloxyfop and clethodim, respectively, compared with the susceptible counterpart. However, ACCase-inhibitor-resistant *A. myosuroides* populations with the Ile-1781-Leu mutation were only resistant to haloxyfop, and not clethodim, regardless of the homozygosity of individuals for this mutation^38^. Thus, the pattern of resistance to ACCase-inhibitors could vary between grass species, notwithstanding the same target site mutation being involved in their mechanism of resistance to ACCase-inhibitors^36^.

Yu *et al*.^39^ suggested that the level of ploidy could affect the level of resistance of a weed species to herbicides. In polyploid species, the level of resistance to a herbicide provided by a mutation could be lower than that for a diploid species if the mutation is only present in one of homoeologous copies of the gene encoding the target enzyme. They suggested that in polyploid species, other homoeologous copies of the gene that do not have the site mutation could still express the wild-type version of the enzyme, thus the effect of the mutation would be diluted. This would result in a herbicide-resistant species showing lower levels of resistance to herbicides if they are polyploid compared with diploid species^39^. The level of ploidy could be the reason why *P. annua* is normally effectively controlled by haloxyfop and clethodim despite the presence of the Ileu-1781-Leu mutation, as the mutation responsible for this amino acid change is present in only one homoeologous copy of the *ACCase* gene^23^, and the other copy still expresses the susceptible ACCase^39^. However, a higher rate of both herbicides would still be needed for controlling *P. annua* compared to other weed species. The recommended label rate for both haloxyfop and clethodim for controlling many other annual grass species such as *Digitaria sanguinalis* and *Echinochloa crus-galli* is only half of the amount needed for controlling *P. annua*^24^.

The translated product of the second PCR primer pair, accf2/accr2, revealed that there is a site mutation at position Ile-2041. At this position, a transversion of thymine to cytosine on the second base of the codon was recorded that resulted in a substitution from isoleucine to threonine. To our knowledge, this mutation has previously been reported only in *A. aequalis*^40^ and now we have found it in haloxyfop-resistant *P. annua*. The ACCase-inhibitor-resistant *A. aequalis* biotype with the Thr-2041 mutation was also found to be sensitive to clethodim, matching our results in this research. Based on R/S GR_50_, the level of resistance to haloxyfop in *A. aequalis* with theThr-2041 mutation was estimated to be 5-fold^37^_,_ whereas the level of resistance to haloxyfop in *P. annua* in our research based on growth reduction was approximately 17-fold. According to Burgos *et al*.^41^, GR_50_ values can be variable and change significantly owing to factors such as environmental conditions or variation in growth, whereas the value for LD_50_ is unlikely to be affected by such factors. Thus, it is preferable to use R/S LD_50_ ratios to compare results across different experiments. Unfortunately, there is no information about the level of resistance to haloxyfop in *A. aequalis* based on the R/S LD_50_ ratios.

Although both asparagine and threonine belong to the same class of amino acids, it is interesting that they cause different levels of resistance to haloxyfop. In this research, *P. annua* with the Thr-2014 mutation was found to be up to 20-times more resistant to haloxyfop compared with the susceptible population. The level of resistance to haloxyfop for biotypes with Thr-2041 appears to be lower than for those biotypes with the Asn-2041 mutation. For instance, in *L. multiflorum*, we found that Asn-2041 resulted in over 400-fold resistance to haloxyfop^42^. The levels of ploidy, as explained above, could partially account for the difference in the level of resistance recorded for Thr-2041 in *P. annua* compared with Asn-2041 recorded in other species; we found that the mutation responsible for the change to threonine at position 2041 was only present in one the homoeologous copies of *ACCase* gene in *P. annua*. The other reason for the difference in the level of resistance to ACCase-inhibitors between Thr-2041 and Asn-2041 mutations could relate to different impacts on conformational changes in the binding site of ACCase-inhibitors in the CT domain of ACCase. The 3-D-modelled structures of the CT domain carrying either Asn-2041 or Thr-2041 revealed that the Ile-2041-Asn substitution results in a major structural change in the CT-binding site of ACCase-inhibitors. In agreement with our results, the 3-D CT domain model for *A. myosuroides* with the Ile-2041-Asn substitution showed a major structural torsion at the herbicide-binding site of ACCase-inhibitors^35^. However, we did not observe any structural changes at the herbicide-binding site for Ile-2041-Thr substitution. The structural changes in the herbicide-binding site resulting from the Asn-2041 mutation would cause a greater reduction in haloxyfop molecules reaching their binding site in the resistant phenotype with the Ile-2041-Asn substitution compared to those phenotypes with the Ile-2041-Thr substitution.

In conclusion, we have evaluated the molecular mechanism of resistance in the first recorded case of haloxyfop-resistant in *P. annua*. The results confirmed the association of a point mutation at position 2041 that replaces isoleucine with threonine. However, this mutation confers a lower level of resistance to haloxyfop in comparison to the Ile-2041-Asn substitution. The haloxyfop-resistant *P. annua* was still susceptible to clethodim, thus this herbicide could be used as a part of a management system for the haloxyfop-resistant *P. annua* studied here. Future studies will involve evaluating the fitness cost^43^ and genetic inheritance^44^ of this Ile-2041-Thr mutation in *P. annua* in order to design effective strategies for managing haloxyfop-resistant *P. annua*.

## Materials and Methods

### Plant material

The response of a resistant population (R) of *P. annua* was compared with a known susceptible population (S) at different rates of haloxyfop. The R population was from a golf course in New Plymouth, New Zealand, where haloxyfop had been used frequently to remove *P. annua* and other grass weeds from a fine fescue turf. A preliminary experiment showed that population R had developed resistance to haloxyfop^25^. The resistant plants of population R were kept in a glasshouse and seeds were collected at maturity. During seeding, plants were exposed to natural light (a day length of 14 h), average daily max/min temperatures of 20.2/19.5 °C, and an average relative humidity (RH) of 55%. Population S was collected from Huatoki Domain in New Plymouth, a site close to the area where the resistant population was collected, but previous applications of any herbicides including haloxyfop at this site were unlikely. The plants from population S were also kept in a glasshouse with average daily max/min temperatures of 22.8/20.5 °C, under natural light (a day length of 14 h), and an average relative humidity of 52%. At maturity, seeds were collected from plants of population S.

### Dose-response experiments

#### Response to haloxyfop

The seeds (n = 20) from populations R and S were placed at a depth of 1 cm in pots (1.2 L) filled with bark, fibre, Pacific Pumice (0.5:0.3:0.2)^45^ plus fertiliser (Woodace)^46^. The pots were kept in a heated glasshouse with automated capillary irrigation at 21.5/18.2 °C under natural light and an average relative humidity (RH) of 50%. *P. annua* plants were treated with haloxyfop (Gallant Ultra, containing 520 g ae L^−1^) at the 2–3 tiller stage, at 0, 15, 30, 60, 120, 240, 480, 960, 1920 and 3840 g ae ha^−1^ plus 0.5% (v/v) of Uptake Spraying Oil using a laboratory track sprayer. The sprayer was calibrated to deliver 230 L ha^−1^ of spray solution at 200 kPa^47^.

In this experiment, pots were arranged in a randomised design and three pot replicates were used per treatment. The number of surviving plants were evaluated 4 weeks after treatment. Subsequently, the aboveground foliage was harvested, oven-dried for 48 h at 80 °C and weighed. This experiment was repeated in time using similar conditions mentioned above.

#### Response to haloxyfop plus malathion

Concurrently with the haloxyfop dose-response experiments, the response of R and S populations to different rates of haloxyfop was evaluated after the plants of both populations were treated with malathion, as previous studies have shown that malathion can overcome some forms of resistance to ACCase-inhibitors in grassy weed species^21,48^. For this research, plants of populations R and S were grown from seeds using the same method and under the same conditions described above. Previous studies showed that treating plants with up to 1000 g ai ha^−1^ of malathion did not have any adverse effects on the treated plants, and that the plants could be treated with malathion prior to herbicide treatments^33^. Thus, when the plants of both R and S populations of *P. annua* were at the 2–3 tiller stage, they were treated with 1000 g ai ha^−1^ of malathion (Fyfanon 440 EW, containing 440 g L^−1^ of malathion) 3 h before applying different rates of haloxyfop as mentioned above. The response of plants to haloxyfop was evaluated 4 weeks after treatment by evaluating the survival and dry weight of treated plants using the method described above. This experiment was repeated twice in time and the environmental conditions for the second run were the same as those for the second run of the haloxyfop experiment described above.

#### Response to clethodim

As discussed earlier, clethodim is another recommended ACCase-inhibitor for controlling *P. annua*. Previous studies have shown that evolving resistance to one class of ACCase-inhibitors might also confer cross-resistance to the other classes of ACCase-inhibitors in the resistant weedy grass species^22,42^. Thus, the response of haloxyfop-resistant and susceptible *P. annua* to different rates of clethodim was evaluated. The plants of both resistant and susceptible phenotypes were grown from seeds using the method mentioned above. When the plants were at the 2–3 tiller stage, they were treated with clethodim (Sequence, containing 240 g L^−1^ clethodim as emulsifiable concentrate) at 0, 3.75, 7.5, 15, 30, 60, 120 and 240 g ae ha^−1^ plus 1% (v/v) of Uptake Spraying Oil using the method described by Ghanizadeh *et al*.^42^. The the average RH was 60% and the average daily max/min temperatures were 19.5/17.5 °C, 4 weeks following herbicide application. This experiment was conducted in a randomised design with three pot replicates for each rate. The response of treated seedlings to different rates of clethodim was evaluated 4 weeks after treatment, using the method described for the haloxyfop experiments. The clethodim experiment was run twice in time. In the second run, the average daily max/min temperatures 4 weeks following herbicide application were 20.5/19.6 °C and the average RH was 50%.

### Partial sequencing of *ACCase* gene

Polymerase chain reaction (PCR) was used to amplify the regions of the *ACCase* gene where potential target site mutations have been reported previously^22^. For this, leaf segments of 1 cm were collected from the youngest fully developed leaves of eight plants from each population. A DNeasy Plant Mini Kit (Qiagen) was used to extract the genomic DNA from leaf material. Two primer pairs, accf1 (5′-TGGTGCTCGGATTGGCATTG-3′)/accr1 (5′-AAGAGGTCCACCAATGTTTGC-3′) and accf2 (5′-CCAGATGACCTTGAAGGTG-3′)/accr2 (5′-TGGATCAAGCCTACCCATGC-3′) were designed to partially amplify the regions of the *ACCase* gene that includes potential mutations that confer resistance to ACCase-inhibitors^13^. The *A. myosuroides* plastidic *ACCase* gene sequence (accession number AJ310767) was used as a reference to design both primer pairs.

The PCR amplification reaction (20 µL) contained template DNA (~10 ng), PCR Master Mix (1 x Phusion High-Fidelity, Thermo Fisher Scientific), 0.5 µM of each primer and dimethyl sulfoxide (3%). The PCR cycling program included denaturation at 98 °C (one cycle of 30 s), denaturation at 98 °C (35 cycles of 10 s), 30 s annealing at 60 °C (for accf2 and accr2 primers) or 62 °C (for accf1 and accr1 primers), 45 s extension at 72 °C, followed by final extension at 72 °C (one cycle of 10 min). The PCR products were then sequenced by Massey University Genomic Centre, and the sequence data were evaluated using the method described by Ghanizadeh *et al*.^42^.

The PCR products were cloned in pUC19 vectors. For this, 1 µg of pUC19 DNA was opened with the *Sma*I restriction enzyme (New England Biolabs) at 25 °C for 1h. Open pUC19 was purified using a QIAquick Gel Extraction Kit (Qiagen) and quantified by NanoDrop (Thermo Scientific, Wilmington, DE, USA). The purified pUC19 was dephosphorylated using rSAP Shrimp Alkaline Phosphatase (New England Biolabs) at 37 °C for 30 min, with the enzyme subsequently inactivated at 65 °C for 5 min according to the manufacturer’s instructions. The PCR products were purified using the method described above and quantified by NanoDrop. The purified PCR products were phosphorylated using T4 polynucleotide kinase (T4 PNK; New England Biolabs) at 37 °C for 30 min, with the enzyme subsequently inactivated at 65 °C for 20 min according to the manufacturer’s instructions. A ligation reaction, based on a 3:1 molar ratio of phosphorylated PCR amplicon:dephosphorylated pUC19 was carried out using T4 DNA ligase overnight at 4 °C as described in the manufacturer’s instructions. The total amount of backbone was set to 50 ng. Half of the ligation reaction was transformed into competent cells of *Escherichia coli* by electroporation using a standard electroporation protocol, and transformants selected on LB agar plates supplemented with 100 µg/ml ampicillin, 80 µg/ml 5-bromo-4-chloro-3-indoyl-β-D-galactopyranoside (X-gal) and 0.1 mM isopropyl β-D-1-thiogalactopyranoside (IPTG) at 37 °C overnight. To determine which colonies carried plasmid with the desired insert, a colony PCR was carried out using Taq DNA polymerase (New England Biolabs) following the manufacturer’s instructions with the accf2 and accr2 primer pair. A 1 min extension and a 60 °C annealing temperature time were used. Plasmid was purified using a QIAprep Spin Miniprep Kit (Qiagen) and sequenced by the Massey Genome Service using the universal M13-F/M13-R primer pair. The DNA sequenced data were analysed using the method described above.

### ACCase protein modelling

Three-dimensional (3-D) structures of the ACCase CT were modelled using the Swiss-model server^49^. Separate models were constructed for each amino acid substitution at position 2041 using yeast (*Saccharomyces cerevisiae*) Protein Data Bank (PDB) accession 1UYT as a template^50^. The CT domain of the ACCase from resistant and susceptible phenotypes was also computed using the 3-D structure of the CT domain of yeast in complex with haloxyfop as a template (PDB accession: 1UYS)^51^ to evaluate the consequences of the amino acid substitutions on the herbicide binding-site. The modelled CT domains were visualised and assessed using Chimera (v.1.13.1).

### Statistical analyses

The data from dose-response experiments were checked for homogeneity of variance (Levene’s tests) and normality (Shapiro-Wilk test) before they were fitted to a three-parameter log-logistic model:

$${\rm{Y}}=[{\rm{d}}/1+\exp ({\rm{b}}\,(\log ({\rm{x}})-\,\log ({{\rm{R}}}_{50})))]$$

where Y is plant survival or dry biomass as a percentage of the untreated control, d is the upper limit, x is herbicide rate, R_50_ is the rate of herbicide corresponding to 50% reduction in plant survival or dry biomass, and b is the slope around R_50_. The data were fitted to this model using the *drc* package in R^52^.

## Supplementary information

Supplementary Information.
