# Supplementary material for: Molecular characteristics of the first case of haloxyfop-resistant Poa annua
Source: Sci Rep. 2020 Mar 6;10:4231. doi: 10.1038/s41598-020-61104-0 (PMC7060245; doi:10.1038/s41598-020-61104-0)
Supplement: Supplementary file 1 — Supplementary Information. [file 41598_2020_61104_MOESM1_ESM.pdf]

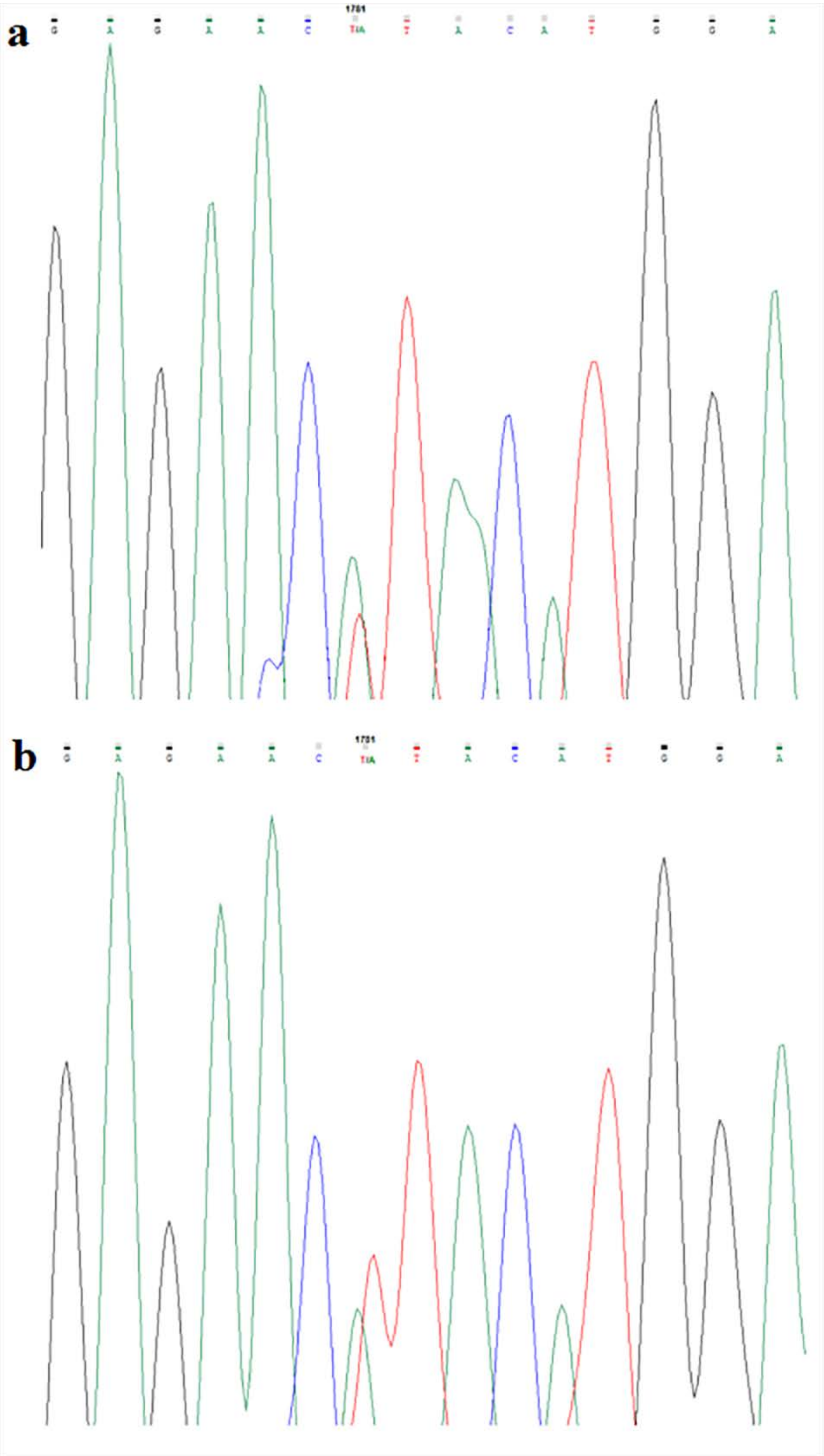

Figure S1. ACCase sequences chromatograms for Ile/Leu-1781 in (a) the haloxyfop-resistant population, R and (b) the haloxyfop-susceptible population, S.

.

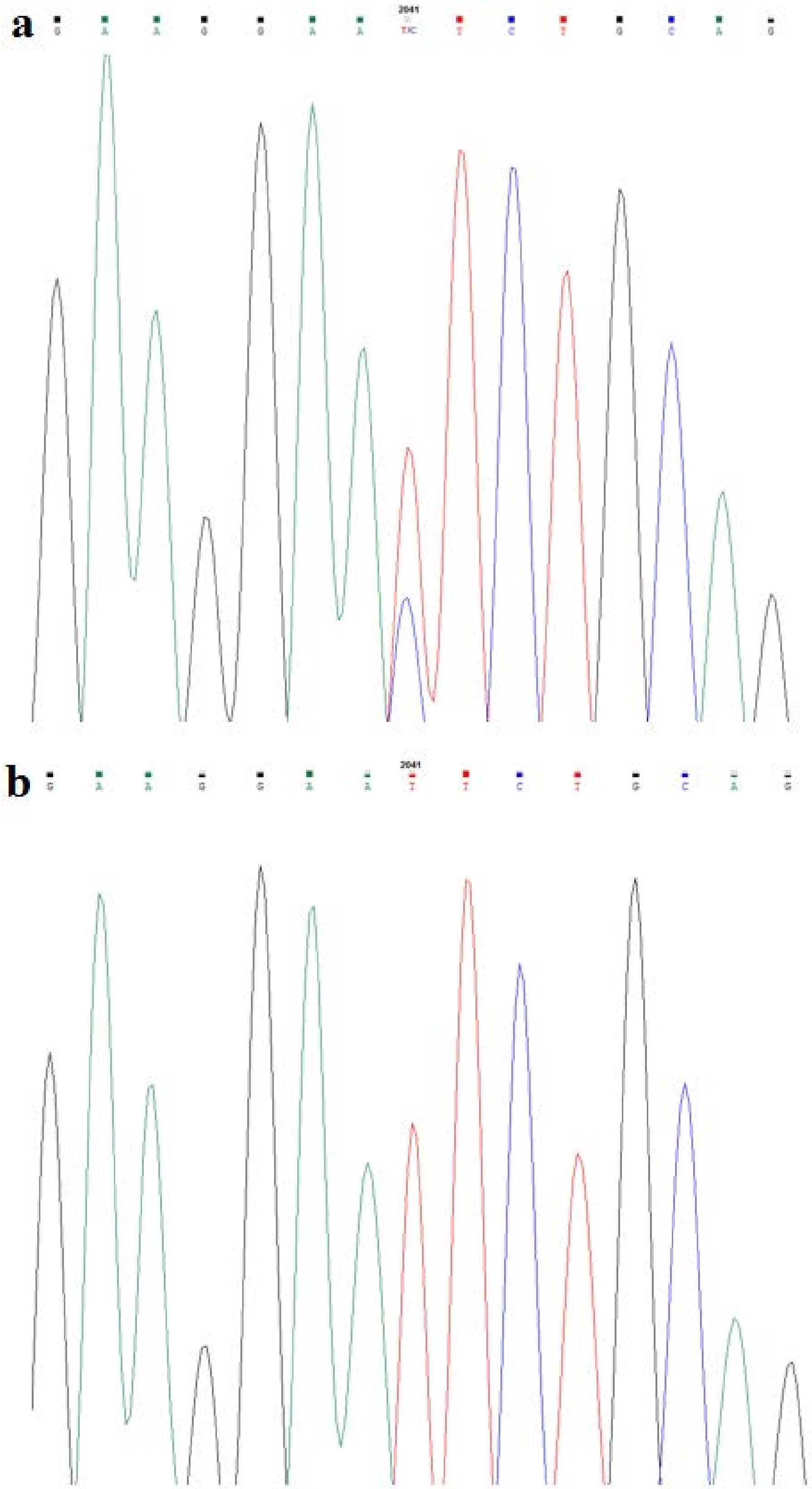

Figure S2. ACCase sequences chromatograms for (a) the ATT/ACT codon for Ile/Thr-2041 in the haloxyfop-resistant population R and (b) the ATT codons for Ile-2041 in the haloxyfop-susceptible population S.

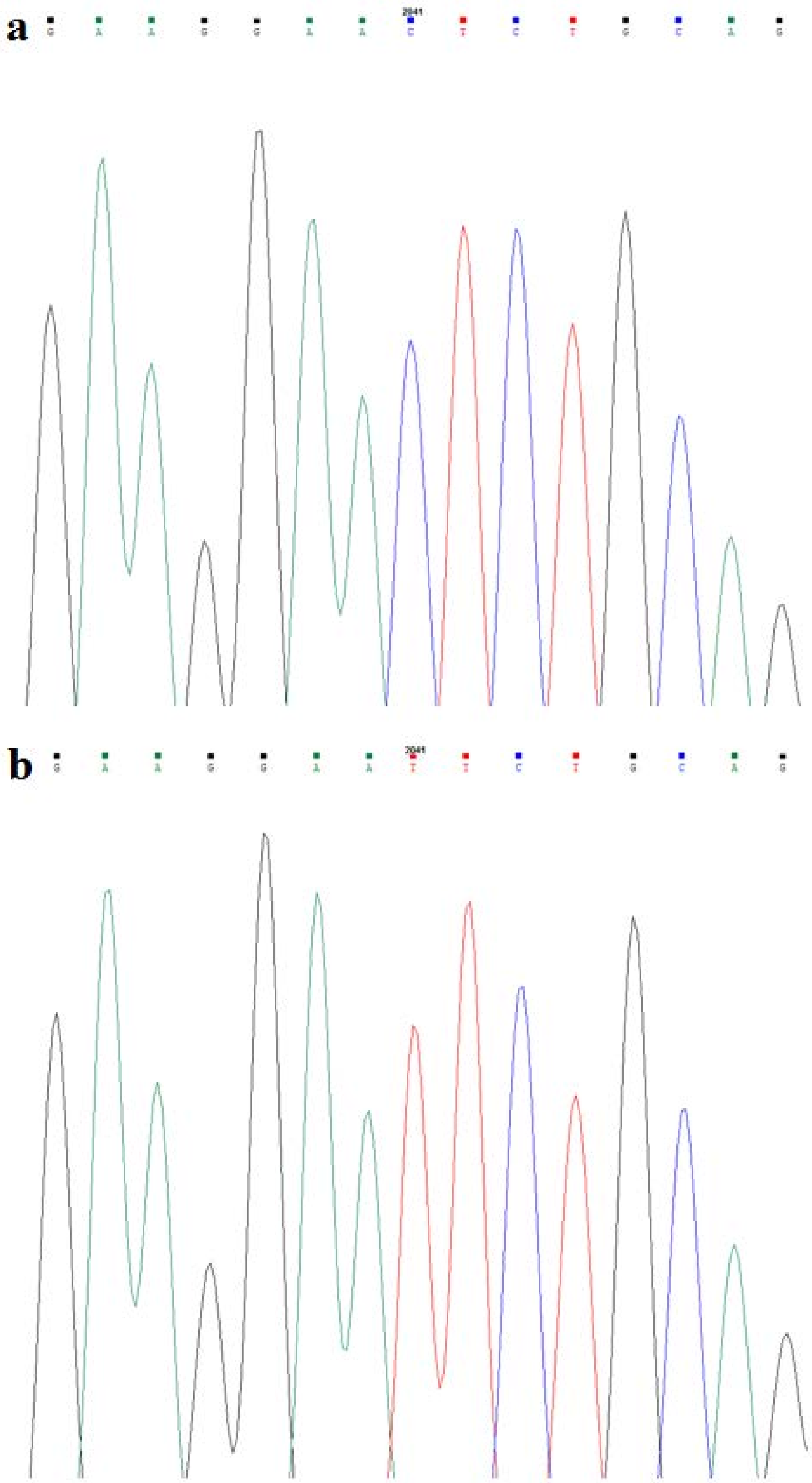

Figure S3. ACCase sequences chromatograms of the haloxyfop-resistant population, R after cloning in pUC19 vectors with (a) the ACT codons for Thr-2041 and (b) the ATT codons for Ile-2041.
